# Association between weight loss agents and elevated liver enzymes: a population-based cross-sectional study

**DOI:** 10.1038/s41598-023-41908-6

**Published:** 2023-09-22

**Authors:** Ye-Jee Kim, Seo Young Kang, Mi-Sook Kim, Joongyub Lee, Bo Ram Yang

**Affiliations:** 1 Department of Clinical Epidemiology and Biostatistics, Asan Medical Center, University of Ulsan College of Medicine, Seoul, Republic of Korea; 2 Department of Family Medicine, Uijeongbu Eulji Medical Center, Eulji University School of Medicine, Uijeongbu, Republic of Korea; 3 Medical Research Collaborating Center, Seoul National University Hospital, Seoul, Republic of Korea; 4 Department of Preventive Medicine, Seoul National University College of Medicine, Seoul, Republic of Korea; 5 College of Pharmacy, Chungnam National University, Daejeon, Republic of Korea

**Keywords:** Public health, Epidemiology

## Abstract

The widespread use of body weight control agents might be related to liver enzyme elevation, but this potential association has only been documented in a few case reports. This study aimed to investigate the associations between weight loss agents and elevated liver enzymes at the population-level. We conducted a cross-sectional study using Korea National Health and Nutrition Examination Survey (KNHANES) data from 2013 to 2019. This study included 36,259 participants over 20 years of age who completed the questionnaire and had no history of hepatitis, cancer, or renal failure. In these participants, we analyzed associations between weight loss agents and elevated liver enzymes by constructing multiple logistic regression models with adjustment for confounding factors and stratified by sex, age, and body mass index. The use of weight loss agents related to liver enzyme elevation in men (adjusted odds ratio (aOR): 1.36, 95% confidence interval (CI): 1.08–1.71) and participants aged less than 40 years (aOR: 1.44, 95% CI: 1.12–1.87). Using more types of weight loss agents was associated with liver enzyme elevation (aOR: 1.31, 95% CI: 1.03–1.67 for 1 weight loss agent, aOR: 1.93, 95% CI: 0.93–3.99 for ≥ 2 weight loss agents). Elevated liver enzymes were associated with the use of traditional medicines (aOR: 1.96, 95% CI: 1.14–3.34) and dietary supplements (aOR: 1.33, 95% CI: 1.02–1.72) in men. We observed an association between weight loss agents and liver enzyme elevation in men, particularly for traditional herbal medicines and dietary supplements. To confirm the observed associations, studies higher on the evidence hierarchy are needed.

## Introduction

Obesity represents a significant challenge in the prevention of chronic diseases and poses a global public health threat. According to the 2020 Korea National Health and Nutrition Examination Survey (KNHANES), the prevalence of obesity, defined as a body mass index (BMI) over 25 kg/m^2^, was 48.0% in men and 27.7% in women over the age of 20^1^. Managing obesity involves lifestyle changes such as exercise and diet modifications, as well as pharmacological treatments including appetite suppressants, lipolysis inhibitors, and glucagon-like peptide 1 receptor agonists. Non-prescription drugs, dietary supplements, and traditional herbal medicines are also commonly used for body weight control due to their perceived effectiveness and convenience^2^. The use of dietary supplements as a strategy for weight control has become more common, increasing from 3% to 6.2% in men and from 3.2% to 8.7% in women from 2007 to 2017, according to KNHANES data^3^.

The incidence of liver injuries linked to traditional herbal medicine and dietary supplements has emerged as a significant public health concern^4^, particularly in Asian countries^5,6^. A prospective nationwide study in Korea, which included 321 cases of drug-induced liver injury (DILI), found that herbal preparations were implicated in approximately 40% of cases, while dietary supplements were responsible for 13.7% of DILI cases^5^. A similar association was reported in a multi-center prospective study from Taiwan, where out of 1297 cases, 285 (22.0%) of DILI cases were attributed to herbal or dietary supplements^6^.

The United States Food and Drug Administration (US FDA) issued a warning about the risk of severe liver injury associated with weight loss agents including dietary supplements^7,8^ and weight loss medications^9^. However, it is important to note that these safety communications were based on case report reviews, indicating a need for larger-scale evaluations. Thus, we aimed to explore the associations between weight loss agents and elevated liver enzyme levels at the population level using the KNHANES database.

## Results

Participants’ baseline characteristics according to their weight control methods are presented in Table 1. On average, participants who tried to lose or maintain weight using weight loss agents were younger (43.4 ± 13.9 years) than those who engaged in no weight control or gained weight (53.3 ± 17.7 years) and those who tried to lose or maintain weight with other methods (48.8 ± 15.5 years). The proportion of women was higher among participants who tried to lose or maintain weight using weight loss agents. The survey year, household income level, occupational type, marital status, education, degree of perceived stress, obesity, smoking status, alcohol consumption, and presence of comorbidities showed significant differences between groups (Table 1).

**Table 1:** Baseline characteristics of participants by weight control method in KNHANES 2013 − 2019.

	Weight control method			*p*-value
	No control or gained weight	Tried to lose or maintain weight		
		Other methods	Weight loss agent	
N	14,599	19,088	2572	
Age, mean ± SD	53.3 ± 17.7	48.8 ± 15.5	43.4 ± 13.9	< 0.001
Sex, n (%)				< 0.001
Male	7346 (50.32)	8112 (42.50)	498 (19.36)	
Female	7253 (49.68)	10,976 (57.50)	2074 (80.64)	
Survey year, n (%)				< 0.001
2013	1788 (12.25)	2605 (13.65)	273 (10.61)	
2014	1808 (12.38)	2404 (12.59)	270 (10.50)	
2015	1858 (12.73)	2611 (13.68)	315 (12.25)	
2016	2253 (15.43)	2856 (14.96)	363 (14.11)	
2017	2265 (15.51)	2878 (15.08)	379 (14.74)	
2018	2364 (16.19)	2813 (14.74)	485 (18.86)	
2019	2263 (15.50)	2921 (15.30)	487 (18.93)	
Household income level, n (%)				< 0.001
Low	3602 (24.80)	2685 (14.11)	218 (8.51)	
Medium–low	3745 (25.79)	4580 (24.07)	638 (24.89)	
Medium–high	3727 (25.66)	5416 (28.46)	815 (31.80)	
High	3449 (23.75)	6347 (33.36)	892 (34.80)	
Occupational type, n (%)				< 0.001
Unemployment or economically inactive persons	5532 (37.89)	6908 (36.19)	864 (33.59)	
Non-manual workers	2753 (18.86)	5155 (27.01)	770 (29.94)	
Manual workers	4002 (27.41)	3874 (20.30)	350 (13.61)	
Service or sales workers	1570 (10.75)	2491 (13.05)	499 (19.40)	
Unknown/No response	742 (5.08)	660 (3.46)	89 (3.46)	
Marital status, n (%)				< 0.001
Yes	12,255 (83.94)	15,690 (82.20)	2027 (78.81)	
No	2342 (16.04)	3397 (17.80)	545 (21.19)	
Education, n (%)				< 0.001
≤ Middle school	5055 (34.63)	4227 (22.14)	346 (13.45)	
High school	3522 (24.12)	5340 (27.98)	785 (30.52)	
≥ College	5275 (36.13)	8869 (46.46)	1353 (52.6)	
Degree of perceived stress, n (%)				< 0.001
Extremely	702 (4.81)	750 (3.93)	199 (7.74)	
Much	3113 (21.32)	3988 (20.89)	704 (27.37)	
Slightly	7908 (54.17)	11,424 (59.85)	1419 (55.17)	
Scarcely	2844 (19.48)	2910 (15.25)	248 (9.64)	
Unknown/no response	32 (0.22)	16 (0.08)	2 (0.08)	
BMI, mean ± SD	23.0 ± 3.4	24.5 ± 3.4	25.1 ± 4.0	< 0.001
Obesity, n (%)				< 0.001
Yes (BMI ≥ 25 kg/m^2^)	3491 (24.00)	7743 (40.62)	1143 (44.51)	
No (BMI < 25 kg/m^2^)	11,057 (76.00)	11,320 (59.38)	1425 (55.49)	
Smoking status, n (%)				< 0.001
Non-smoker	7980 (54.66)	11,952 (62.62)	1786 (69.44)	
Ex-smoker	3029 (20.75)	4085 (21.40)	421 (16.37)	
Current smoker	3563 (24.41)	3034 (15.89)	362 (14.07)	
Unknown/no response	27 (0.18)	17 (0.09)	3 (0.12)	
Alcohol consumption, n (%)				< 0.001
Heavy drinker	1736 (11.89)	2127 (11.14)	330 (12.83)	
Light-to-moderate drinker	8349 (57.19)	12,396 (64.94)	1758 (68.35)	
Non-drinker	4500 (30.82)	4558 (23.88)	483 (18.78)	
Unknown/no response	14 (0.1)	7 (0.04)	1 (0.04)	
Medium intensity physical activity, n (%)				< 0.001
Yes	968 (6.63)	1406 (7.37)	219 (8.51)	
No	11,121 (76.18)	14,436 (75.63)	1990 (77.37)	
Unknown/no response	2510 (17.19)	3246 (17.01)	363 (14.11)	
Comorbidities, n (%)				
Hypertension, n (%)				< 0.001
Yes	3648 (24.99)	4140 (21.69)	356 (13.84)	
No	10,772 (73.79)	14,719 (77.11)	2185 (84.95)	
Unknown/no response	179 (1.23)	229 (1.20)	31 (1.21)	
Hyperlipidemia, n (%)				< 0.001
Yes	2276 (15.59)	3280 (17.18)	357 (13.88)	
No	12,144 (83.18)	15,579 (81.62)	2184 (84.91)	
Unknown/no response	179 (1.23)	229 (1.20)	31 (1.21)	
Diabetes mellitus, n (%)				< 0.001
Yes	1463 (10.02)	1528 (8.01)	133 (5.17)	
No	12,956 (88.75)	17,329 (90.78)	2408 (93.62)	
Unknown/no response	180 (1.23)	231 (1.21)	31 (1.21)	
Depressive disorder, n (%)				< 0.001
Yes	631 (4.32)	771 (4.04)	153 (5.95)	
No	13,294 (91.06)	17,702 (92.74)	2334 (90.75)	
Unknown/no response	674 (4.62)	615 (3.22)	85 (3.30)	
Thyroid disease, n (%)				< 0.001
Yes	439 (3.01)	676 (3.54)	115 (4.47)	
No	13,487 (92.38)	17,797 (93.24)	2372 (92.22)	
Unknown/no response	673 (4.61)	615 (3.22)	85 (3.3)	
Family history of HBV infection, n (%)				< 0.001
Yes	215 (1.47)	366 (1.92)	55 (2.14)	
No	10,784 (73.87)	14,650 (76.75)	2067 (80.37)	
Unknown/no response	3600 (24.66)	4072 (21.33)	450 (17.5)	

*KNHANES* Korean National Health and Nutrition Examination Survey; *SD* standard deviation; *BMI* body mass index; *HBV* hepatitis B virus.

Compared to those who engaged in no weight control, participants who used weight loss agents showed a non-statistically significant higher prevalence of elevated liver enzymes (adjusted odds ratio, aOR: 1.11, 95% confidence interval (CI): 0.95–1.29). The type of weight loss agents did not show significant associations with elevated liver enzymes (Table 2).

**Table 2:** Associations between weight control method and elevated liver enzymes.

Type of weight control methods	Total	No. of events (%)	Crude OR (95% CI)	*p*-value	Adjusted OR (95% CI)	*p*-value
Weight control attempts						
No control or gained weight	14,599	1305 (8.94)	Reference		Reference	
Tried to lose or maintain weight						
Other methods	19,088	1911 (10.01)	1.13 (1.05, 1.22)	< 0.001	0.94 (0.87, 1.02)	0.137
Weight loss agent	2572	252 (9.8)	1.11 (0.96, 1.28)	0.162	1.11 (0.95, 1.29)	0.195
Types of weight loss agents						
No control or gained weight	14,599	1305 (8.94)	Reference		Reference	
Tried to lose or maintain weight						
Weight loss medications without prescription	233	27 (11.59)	1.34 (0.89, 2.00)	0.162	1.28 (0.83, 1.96)	0.261
Weight loss medications with prescription	682	60 (8.80)	0.98 (0.75, 1.29)	0.899	1.11 (0.83, 1.48)	0.488
Traditional herbal medicine	474	41 (8.65)	0.96 (0.7, 1.34)	0.828	0.95 (0.68, 1.34)	0.788
Dietary supplements	1570	163 (10.38)	1.18 (0.99, 1.4)	0.059	1.14 (0.95, 1.37)	0.163
Combination of weight loss agents						
No control or gained weight	14,599	1305 (8.94)	Reference		Reference	
Tried to lose or maintain weight						
Other methods	19,088	1911 (10.01)	1.13 (1.05, 1.22)	< 0.001	0.94 (0.87, 1.02)	0.138
Weight loss agent (1 type)	2227	218 (9.79)	1.11 (0.95, 1.29)	0.193	1.1 (0.93, 1.29)	0.261
Weight loss agent (≥ 2 types)	345	34 (9.86)	1.11 (0.78, 1.59)	0.556	1.18 (0.81, 1.72)	0.398

*OR* odds ratio, *CI* confidence interval.

Adjusted for age, sex, survey year, occupational type, marital status, education, degree of perceived stress, obesity, smoking status, alcohol consumption, medium-intensity physical activity, hypertension, hyperlipidemia, diabetes mellitus, thyroid disease and the presence of family history of hepatitis B virus infection.

**Figure 1:**
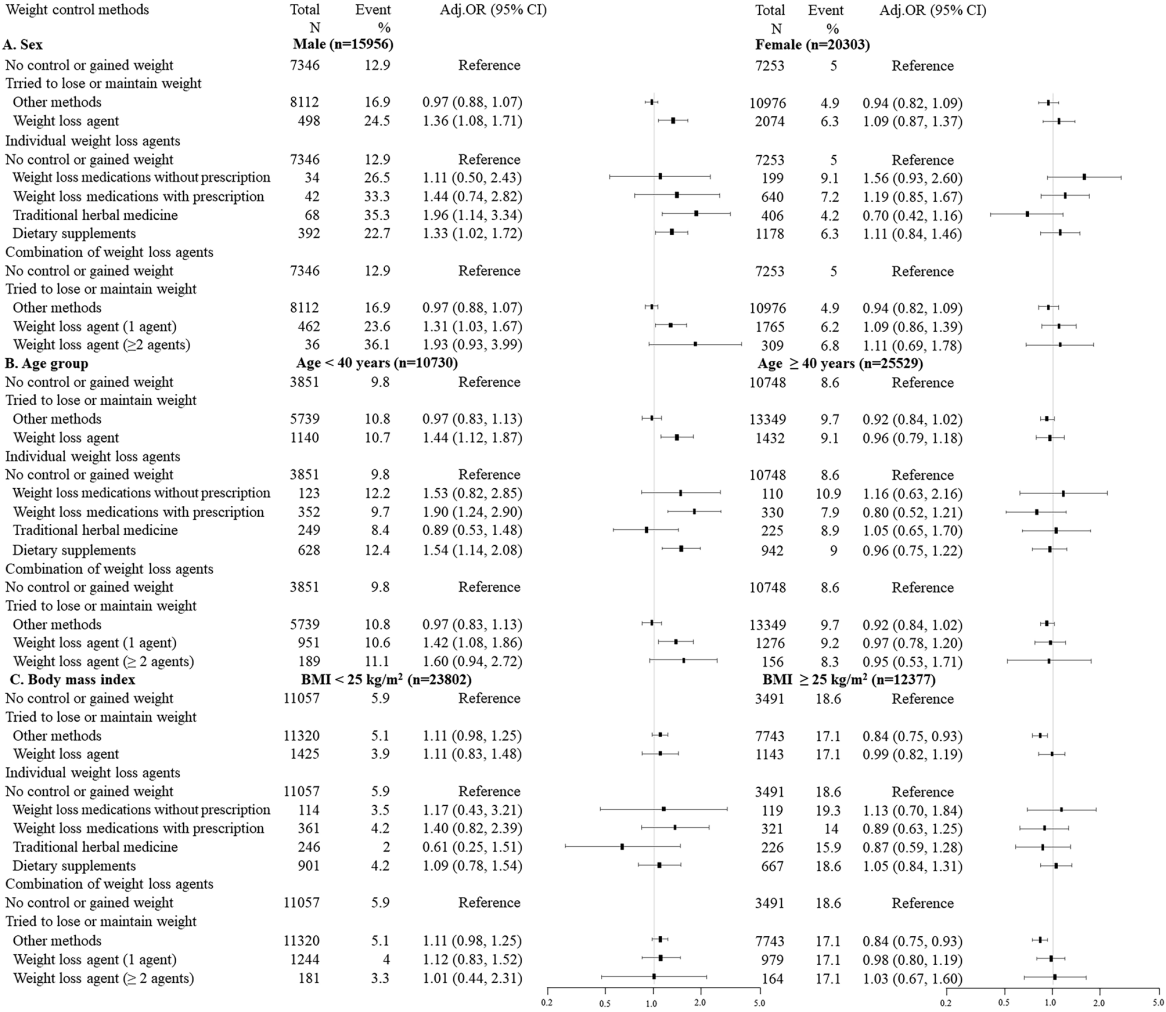
Associations between weight control method and elevated liver enzymes, with results stratified according to sex, age group and body mass index. *OR* odds ratio; *CI* confidence interval. Adjusted for age, sex, survey year, occupational type, marital status, education, degree of perceived stress, obesity, smoking status, alcohol consumption, medium-intensity physical activity, hypertension, hyperlipidemia, diabetes mellitus, thyroid disease and the presence of family history of hepatitis B virus infection.

Figure 1A shows stratified results according to sex. Among 15,956 male participants, 498 (3.12%) took weight loss agents, whereas 10.2% of female participants (n = 2074) used weight loss agents. An association between weight loss agent use and elevated liver enzymes was observed in men (aOR: 1.36, 95% CI: 1.08–1.71), but not women (aOR: 1.09, 95% CI 0.87–1.37). Regarding the risks for the types of weight loss agents, women using weight loss medications without prescriptions showed a higher tendency of elevated liver enzymes, but not significant (aOR: 1.56, 95% CI: 0.93–2.60). For men, elevated risks were found in association with the use of traditional medicines (aOR: 1.96, 95% CI 1.14–3.34) and supplements (aOR: 1.33, 95% CI: 1.02–1.72). In men, elevated liver enzymes appeared to be related to the number of weight loss agents used. The aOR was 1.31 (95% CI: 1.03–1.67) in participants using one type of weight loss agent, and 1.93 (95% CI: 0.93–3.99) in participants using two or more types of weight loss agents (Fig. 1A).

Figure 1B shows the associations of elevated liver enzymes with weight loss agents according to the age group. Participants aged less than 40 years old using weight loss agents showed increased risks of elevated liver enzymes (aOR: 1.44, 95% CI: 1.12–1.87), and significant associations were found between elevated liver enzymes and the use of weight loss medications with prescription (aOR: 1.90, 95% CI:1.24–2.90) and supplements (aOR: 1.54, 95% CI 1.14–2.08). An increasing trend in the prevalence of elevated liver enzymes according to the number of weight loss agent types was observed (aOR: 1.42, 95% CI: 1.08–1.86 for 1 weight loss agent, aOR: 1.60, 95% CI: 0.94–2.72 for ≥ 2 weight loss agents). However, increased risk estimates of elevated liver enzymes were not observed among participants aged over 40.

Figure 1C shows BMI-stratified results for elevated liver enzymes according to the weight loss agents. Although increased point estimates of ORs were observed in participants with a BMI < 25 kg/m^2^, neither stratum showed a statistically significant increase in elevated liver enzymes.

**Figure 2:**
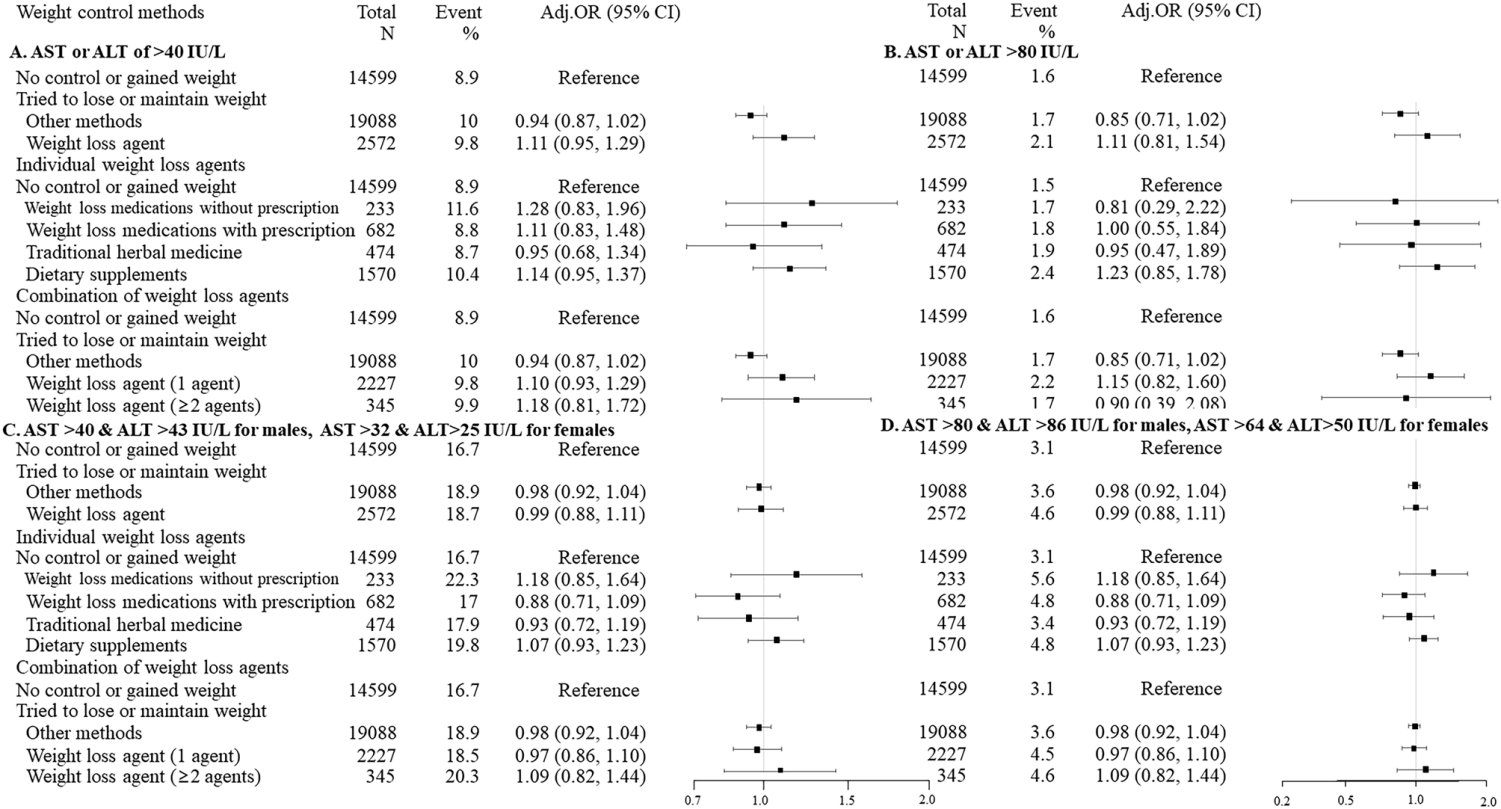
Sensitivity analyses according to several cut-offs for liver enzyme elevation. OR, odds ratio; CI, confidence interval. Adjusted for age, sex, survey year, occupational type, marital status, education, degree of perceived stress, obesity, smoking status, alcohol consumption, medium-intensity physical activity, hypertension, hyperlipidemia, diabetes mellitus, thyroid disease and the presence of family history of hepatitis B virus infection.

The results were robust in sensitivity analyses according to several cut-offs of alanine aminotransferase (ALT) and aspartate aminotransferase (AST) for liver enzyme elevation (Fig. 2).

A sensitivity analysis assessing the association between weight loss and liver enzyme levels that treated AST and ALT as continuous variables also yielded results that were consistent with the findings from multivariable logistic regression, especially regarding the analysis of ALT as a continuous variable (Supplementary Table 1, 2).

## Discussion

Our study investigated the association of weight loss agent use with elevated liver enzymes using a nationwide population-based cross-sectional database. Elevated liver enzymes were associated with the use of weight loss agents in men and young participants, and the number of weight loss agent types used was also associated with increased risk. Of particular note, elevated liver enzymes were associated with the use of traditional medicine and dietary supplements in men, and with weight loss medication use in young participants.

The association between the use of traditional herbal medicines and elevated liver enzymes was significant in men. The majority of cases of herbal-induced liver injury are due to multi-ingredient, and the component responsible for the toxicity is usually unknown or can only be suspected^10^. Specific ingredients for traditional herbal medicine were not identified in the KNHANES database we used; however, Kim et al. reported frequently studied traditional medicines for obesity from 2015 to 2019 in Korea, and most frequently studied herbs were *Ephedrae herba* and *Glycyrrhizae radix,* and the herbal formulas were *taeeumjowi-tang (taiyindiaowei-tang), bangpungtongsung-san (fangfengtongsheng-san),* and *yanggyeoksanhwa-tang (lianggesanhuo-tang)*^11^. Products containing ephedra or ephedrine alkaloids, which are often marketed for weight loss in Asian countries, have been linked to adverse liver-related events, including acute and autoimmune hepatitis^12^. From human hepatic cell study, it was found that Ephedrine induced mitochondrial oxidative stress and depolarization and inhibiting mitochondrial biogenesis. These combined effects led to liver cell death through imbalance of autophagic flux^13^. Despite the known safety risks associated with liver injuries, traditional herbal medicine is often viewed by the public as a safe healthcare option due to its long history of use^14^. However, the regulatory system for traditional medicine and supplements does not require a detailed safety profile, and unlike conventional drugs, post-marketing surveillance is not actively conducted^15^. There is a need for patient education regarding the potential risks of liver injury associated with traditional medicine, as well as initiatives to increase healthcare professionals' awareness of liver injuries induced by traditional medicine.

In our study, traditional herbal medicine for weight loss were associated with an elevated liver enzyme especially in male participants. Although there has been limited research conducted on the presence of sex-differences in adverse event associated with traditional herbal medicine, a systematic review reported that male sex was significantly associated with an higher risk of death or liver transplantation in patients with liver injury induced by traditional Chinese medicine^16^. However, elevated liver enzyme levels can occur due to extrahepatic causes, such as vigorous exercise, and muscular injuries may lead to elevated transaminase levels. Weight loss agent users may be more likely to participate in muscular exercise, which could be associated with liver enzyme elevation^17^. Further studies are needed to confirm the association of traditional herbal medicine use and elevated liver enzyme in male.

The use of dietary supplements has been significantly associated with men and younger individuals. Garcinia cambogia extract and green tea extract are frequently used as dietary supplements for reducing body fat, particularly among Korean university students^18^. A study analyzing adverse events in the Korean dietary supplement database from 2006 to 2018 found that 4.2% of all cases were liver-related adverse events. Notably, Garcinia cambogia extract and green tea extract were among the top five most frequently reported ingredients in dietary supplements. An in vitro study observed liver toxicity in response to combined Garcinia cambogia extract and green tea extract, as determined by an AST/ALT activity test^19^. There have been several case reports and case-series studies of liver injury following the use of Garcinia cambogia extract for weight loss in the US, Asia, and Europe^4^. In 2009, the US FDA issued a warning related to hepatotoxicity induced by Hydroxycut™, a supplement based on Garcinia cambogia extract^8^. Green tea extract is also considered a potential culprit of liver injury, as evidenced by several case reports^10^. Regulatory agencies in the US and Korea have recommended setting a limit on the daily intake of green tea extracts to prevent serious liver damage^7,20^.

While no statistically significant association was observed in the overall study population, the association between the use of weight loss agents without prescription and the risk of elevated liver enzymes appeared to be stronger in women. While previous studies have not specifically investigated sex differences in the adverse event of weight loss agents, it is widely recognized that women generally have a 1.5–1.7 times higher risk of drug-related adverse events^21^. Furthermore, women were more susceptible to DILI than men, which might be explained by sex differences in pharmacokinetics including factors such as body weight, body fat composition, and hormonal effects^22^.

Our study has several strengths. We used a nationally representative sample from a 7-year period, which permitted the generalization of the results to the overall population of South Korea. With detailed information on weight control methods, objective measurements of liver enzyme levels, and participants’ characteristics, it was possible to obtain meaningful results regarding the effects of weight control methods on elevated liver enzymes. We identified consistent results when adjusting for potential confounders, stratifying participants, and analyzing liver enzyme levels as continuous variables.

However, the present study should be interpreted considering several limitations. First, the temporal relationship between weight control methods and elevated liver enzymes could not be determined since this is a cross-sectional study. To confirm the observed associations, studies higher on the evidence hierarchy, such as cohort studies employing a new user design satisfying temporal relationships, are needed. Second, the information on weight control methods was collected by a self-reported questionnaire, and misclassification could have taken place due to recall bias. However, non-differential recall bias might have occurred with respect to liver function. Third, the KNHANES dataset had limited information on drug use, preventing us from considering the impact of drugs that could elevate liver enzymes, such as anti-tuberculosis drugs, anti-fungal drugs, and anti-epileptic drugs^23,24^. Finally, the database used in this study did not provide detailed information on the ingredients and usage patterns (dose, duration, etc.) of weight loss agents, making it impossible to conduct a more detailed analysis focusing on each ingredient.

This study revealed an association between the use of weight loss agents and elevated liver enzymes, with variations observed based on age, sex, and BMI. Given the societal pressure to lose weight and the public health implications of obesity, the use of weight loss agents has risen over time. To ensure the safe use of these agents, our study underscores the importance of monitoring serum liver enzymes. This allows the early detection and prompt management of potential liver injuries in adults.

## Methods

### Data source and study subjects

We conducted a cross-sectional study using data from the sixth wave (2013–15), seventh wave (2016–18), and eighth wave (2019) of the KNHANES conducted by the Korea Disease Control and Prevention Agency of the Ministry of Health and Welfare. All participants were selected using multi-stage clustered probability sampling and comprised a representative sample of non-institutionalized civilians in South Korea. All participants gave written informed consent before study participation^23,25^.

**Figure 3:**
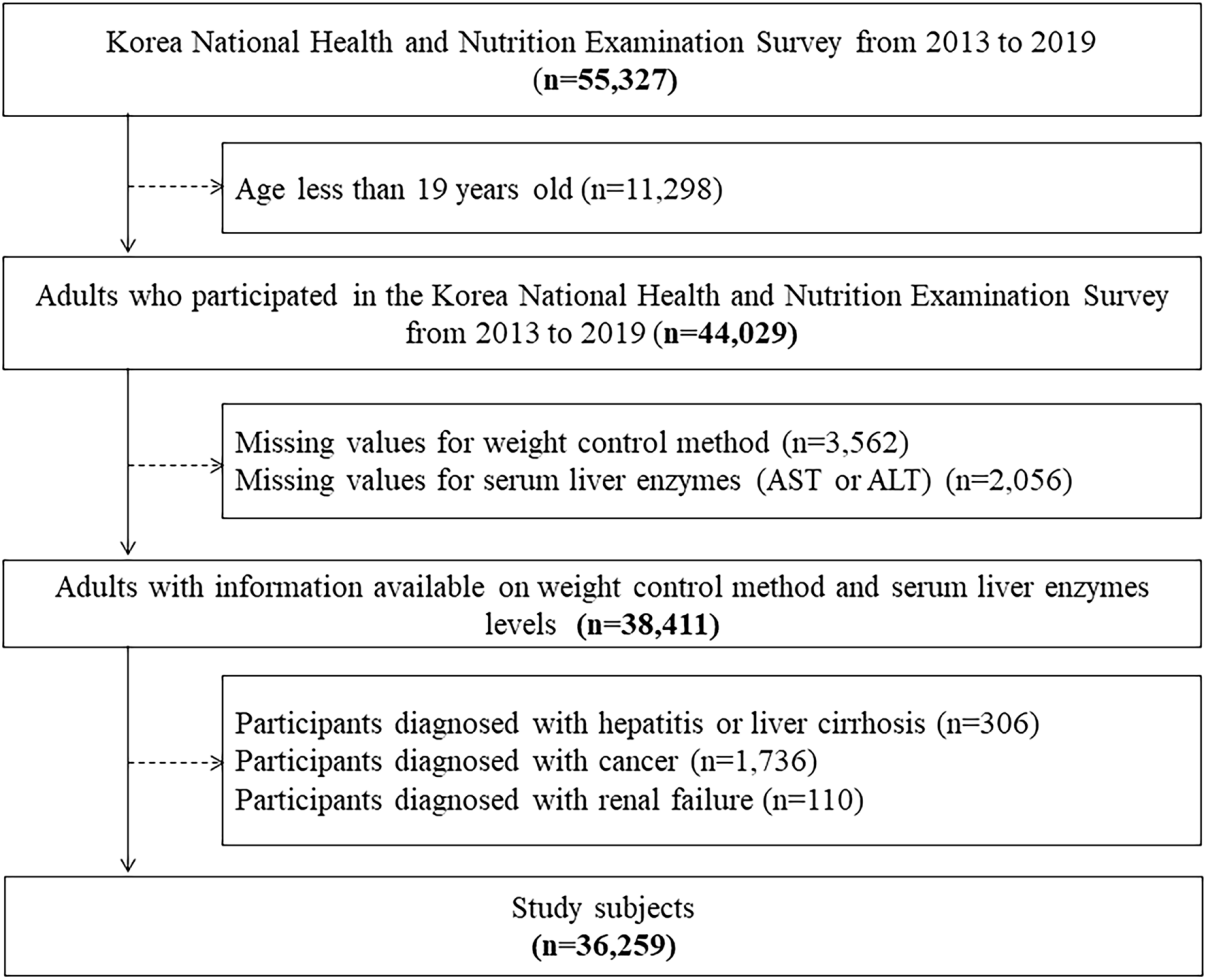
Study flow. *AST* aspartate transaminase; *ALT* alanine transferase.

A total of 55,327 participants completed the KNHANES questionnaire between 2013 and 2019. Of these, we included 44,029 adults aged 19 and older. We excluded 5,618 respondents who lacked liver enzyme test information and had completed a weight control questionnaire. Additionally, we excluded 306 respondents diagnosed with hepatitis B, hepatitis C, or liver cirrhosis. We also excluded 1,736 respondents with a history of various cancers, including stomach, liver, colorectal, breast, cervical, lung, thyroid, and others. Finally, we excluded 110 respondents diagnosed with renal failure (Fig. 3).

### Ethics

This study complies with the Declaration of Helsinki, and the requirement for ethical review and approval was waived for this study by the Asan Medical Center Institutional Review Board (2021–1666), because the KNHANES database contains anonymized data that cannot allow patients’ identification. All participants gave written informed consent before study participation. However, the need for written or verbal consent was waived due to the observational nature of the study and the fact that the patient identifiers were fully encrypted prior to data analysis.

### Weight control methods

We extracted data on participants' experiences with weight management and the strategies they employed over a 1-year period. The data were collected by administering a questionnaire that asked: "Have you made any conscious efforts to manage your weight in the past year?" and "Please list all the methods you used to either lose or maintain your weight over the past year." We recorded attempts at weight control, including no control, efforts to gain weight, and efforts to lose or maintain weight. Only participants who indicated they had tried to lose or maintain weight answered the subsequent question. We documented the weight control methods, which included exercise, fasting, reducing food intake, skipping meals, using prescription weight-loss drugs, using over-the-counter weight-loss drugs, using herbal medicine, consuming functional foods, and adhering to a single-food diet. We classified weight control methods into two categories: the use of weight loss agents (prescription and non-prescription weight loss medication, traditional herbal medicine, and dietary supplements), and other methods (exercise, fasting, reducing food intake, skipping meals, single-food diet, and so on)^3^.

### Liver enzymes

Liver aminotransferases (ALT and AST) are markers commonly used in routine serum liver enzyme tests. Levels of these enzymes increase when hepatocytes are damaged under disease conditions such as hepatitis, cholestasis, severe steatosis, and others. ALT and AST levels were obtained from venous blood samples and measured using the Hitachi Automatic Analyzer 7600–210 (Hitachi, Tokyo, Japan). The measurement was conducted using the International Federation of Clinical Chemistry method, which employs ultraviolet light without pyridoxal-5′-phosphate, and utilized the Pureauto S series reagent (Sekisui, Tokyo, Japan)^25,26^. Although a lower cut-off has been suggested to define an elevated status of AST or ALT, we primarily classified elevated liver enzymes using the traditional cut-off levels of > 40 IU/L^27^. Sensitivity analyses of several cut-offs were conducted to investigate the robustness of the results as follows: 1) American College of Gastroenterology Clinical Guideline (ACG CG): AST > 43 IU/L and ALT > 33 IU/L for males, AST > 32 IU/L and ALT > 25 IU/L for females, 2) higher than borderline elevation defined using 2 times the upper limit of normal (ULN) of the traditional cut-off level: AST or ALT > 80 IU/L, and 3) higher than borderline elevation using 2 times the ULN of the ACG CG: AST > 80 IU/L and ALT > 86 IU/L for males, AST > 64 IU/L and ALT > 50 IU/L for females^28^.

### Variables

To comprehensively evaluate potential confounding factors and account for their effects, we incorporated demographics, behavioral factors, comorbidities, and family history into our analysis. The demographic variables we considered included age, sex, survey year, household income (categorized as low, medium–low, medium–high, or high), occupational type (classified as unemployed or economically inactive, non-manual workers, manual workers, service or sales workers, or unknown/no response), marital status (either married or not), and educational status (grouped as middle school or less, high school, or college and above). Behavioral factors encompassed stress recognition levels (rated as high, moderate, low, or unknown/no response), BMI, obesity, smoking status (categorized as non-smoker, ex-smoker, current smoker, or unknown/no response), alcohol consumption (classified as heavy, light-to-moderate, non-drinker, or unknown/no response), and participation in medium-intensity physical activity (either yes, no, or unknown/no response). We defined obesity as a BMI of 25 kg/m^2^ or higher, in accordance with the Asia–Pacific criteria set by the World Health Organization guidelines^29^. We also took into account the participants' history of comorbidities, which included hypertension, hyperlipidemia, diabetes, depressive disorders, and thyroid disease. Furthermore, we collected information on family history of hepatitis B virus infection, as it was deemed a relevant confounding factor in the analysis.

### Statistical analysis

Data for continuous variables are presented as means ± standard deviation (SD), and data for categorical variables are presented as the number of cases with a percentage. Analysis of variance and the chi-square test were used to examine differences between weight control methods. We analyzed associations between elevated liver enzymes and weight control methods using logistic regression models. To adjust for confounders, we selected variables that demonstrated statistical differences between exposure groups. We then conducted logistic regression to identify statistically significant risk factors for elevated liver enzymes, using a *p*-value of < 0.1 as the threshold for significance. We also stratified our data by sex, age group (either under 40 years or 40 years and above), and BMI (non-obese < 25 kg/m^2^, obese ≥ 25.0 kg/m^2^), because the susceptibility to elevated liver enzymes and attempts at weight control vary according to sex, age, and BMI^30–32^. We also performed a sensitivity analysis where AST and ALT were treated as continuous variables using a linear regression model. All statistical analyses were performed with SAS version 9.4 (SAS Institute Inc., Cary, NC, USA). A *p*-value < 0.05 was considered to indicate statistical significance.

### Supplementary Information

Supplementary Tables.

## Data Availability

The data used in this study are openly available from the Korea National Health and Nutritional Examination Survey webpage (URL: https://knhanes.kdca.go.kr/knhanes/sub03/sub03_02_05.do).
