# Author Correction: Association between weight loss agents and elevated liver enzymes: a population‑based cross‑sectional study

**DOI:** 10.1038/s41598-023-47158-w

**Published:** 2023-11-22

**Authors:** Ye‑Jee Kim, Seo Young Kang, Mi‑Sook Kim, Joongyub Lee, Bo Ram Yang

**Affiliations:** 1 Department of Clinical Epidemiology and Biostatistics, Asan Medical Center, University of Ulsan College of Medicine, Seoul, Republic of Korea; 2 Department of Family Medicine, Uijeongbu Eulji Medical Center, Eulji University School of Medicine, Uijeongbu, Republic of Korea; 3 Medical Research Collaborating Center, Seoul National University Hospital, Seoul, Republic of Korea; 4 Department of Preventive Medicine, Seoul National University College of Medicine, Seoul, Republic of Korea; 5 College of Pharmacy, Chungnam National University, Daejeon, Republic of Korea

Correction to: *Scientific Reports* https://doi.org/10.1038/s41598-023-41908-6, published online 22 September 2023

The original version of this Article contained errors in the Results and Discussion sections.

In the Results section, the sentence:

“Participants aged less than 40 years old using weight loss agents showed increased risks of elevated liver enzymes (aOR: 1.44, 95% CI: 1.12–1.87), and significant associations were found between elevated liver enzymes and the use of traditional medicines (aOR: 1.90, 95% CI:1.24–2.90) and supplements (aOR: 1.54, 95% CI 1.14–2.08).”

now reads:

“Participants aged less than 40 years old using weight loss agents showed increased risks of elevated liver enzymes (aOR: 1.44, 95% CI: 1.12–1.87), and significant associations were found between elevated liver enzymes and the use of weight loss medications with prescription (aOR: 1.90, 95% CI:1.24–2.90) and supplements (aOR: 1.54, 95% CI 1.14–2.08).”

In addition, in the Discussion section, the sentence:

“The association between the use of traditional herbal medicines and elevated liver enzymes was significant in men and young people.”

now reads:

“The association between the use of traditional herbal medicines and elevated liver enzymes was significant in men.”

The original Article has been corrected.

