# Supplementary material for: Association between weight loss agents and elevated liver enzymes: a population-based cross-sectional study
Source: Sci Rep. 2023 Sep 22;13:15796. doi: 10.1038/s41598-023-41908-6 (PMC10517163; doi:10.1038/s41598-023-41908-6)
Supplement: Supplementary file 1 — Supplementary Tables. [file 41598_2023_41908_MOESM1_ESM.pdf]

Supplementary table 1. Linear regression analysis of the associations between the weight control method and log-transformed alanine aminotransferase levels

	Simple regression model			Multiple regression model[a]		
	**Beta coefficient**	**Standard error**	***p*-value**	**Beta coefficient**	**Standard error**	***p*-value**
**Weight control attempts**						
No control or gained weight	Reference			Reference		
Tried to lose or maintain weight						
Other methods	0.039	0.006	<.0001	0.022	0.006	<.0001
Weight loss agent	-0.017	0.012	0.136	0.040	0.011	0.000
**Types of weight loss agents**						
No control or gained weight	Reference			Reference		
Tried to lose or maintain weight						
Weight loss medications without prescription	-0.004	0.035	0.914	0.062	0.032	0.053
Weight loss medications with prescription	-0.075	0.021	0.000	-0.018	0.020	0.359
Traditional herbal medicine	-0.050	0.025	0.043	-0.007	0.023	0.766
Dietary supplements	0.014	0.014	0.307	0.036	0.013	0.007
**Combination of weight loss agents**						
No control or gained weight	Reference			Reference		
Tried to lose or maintain weight						
Other methods	0.039	0.006	<.0001	0.022	0.006	<.0001
Weight loss agent (1 type)	-0.016	0.012	0.209	0.038	0.011	0.001
Weight loss agent (≥2 types)	-0.029	0.030	0.328	0.057	0.026	0.031

[a]Adjusted for age, sex, survey year, occupational type, marital status, education, degree of perceived stress, obesity, smoking status, alcohol consumption, medium-intensity physical activity, hypertension, hyperlipidemia, diabetes mellitus, thyroid disease and the presence of family history of hepatitis B virus infection

Supplementary table 2. Linear regression analysis of the associations between the weight control method and log-transformed aspartate aminotransferase levels

	**Simple regression model**			**Multiple regression model[a]**		
	**Beta coefficient**	**Standard error**	***p*-value**	**Beta coefficient**	**Standard error**	***p*-value**
**Weight control attempts**						
No control or gained weight	Reference			Reference		
Tried to lose or maintain weight						
Other methods	-0.017	0.004	<.0001	-0.001	0.004	0.820
Weight loss agent	-0.060	0.007	<.0001	0.002	0.007	0.794
**Types of weight loss agents**						
No control or gained weight	Reference			Reference		
Tried to lose or maintain weight						
Weight loss medications without prescription	-0.075	0.023	0.001	-0.015	0.022	0.493
Weight loss medications with prescription	-0.089	0.014	<.0001	-0.023	0.013	0.081
Traditional herbal medicine	-0.100	0.016	<.0001	-0.033	0.015	0.031
Dietary supplements	-0.037	0.009	<.0001	0.008	0.009	0.351
**Combination of weight loss agents**						
No control or gained weight	Reference			Reference		
Tried to lose or maintain weight						
Other methods	-0.017	0.004	<.0001	-0.001	0.004	0.819
Weight loss agent (1 type)	-0.057	0.008	<.0001	0.002	0.007	0.782
Weight loss agent (≥2 types)	-0.080	0.019	<.0001	0.000	0.017	0.980

[a]Adjusted for age, sex, survey year, occupational type, marital status, education, degree of perceived stress, obesity, smoking status, alcohol consumption, medium-intensity physical activity, hypertension, hyperlipidemia, diabetes mellitus, thyroid disease and the presence of family history of hepatitis B virus infection
